# Understanding public awareness and knowledge of lung cancer screening practices: a cross-sectional study from the Asir region, Saudi Arabia

**DOI:** 10.3389/fpubh.2025.1678080

**Published:** 2025-11-03

**Authors:** Geetha Kandasamy, Khalid Orayj, Asma M. Alshahrani, Tahani S. Alanazi, Amjad Hmlan, Sitah Alharthi, Hanan Alyami, Arwa Khaled

**Affiliations:** ^1^Department of Clinical Pharmacy, College of Pharmacy, King Khalid University, Abha, Saudi Arabia; ^2^Department of Clinical Pharmacy, College of Pharmacy, Shaqra University, Dawadimi, Saudi Arabia; ^3^Department of Pharmaceutics, College of Pharmacy, Shaqra University, Dawadimi, Saudi Arabia; ^4^Department of Medical and Surgical Nursing, College of Nursing, Princess Nourah Bint Abdulrahman University, Riyadh, Saudi Arabia

**Keywords:** lung cancer, knowledge, screening, public awareness, risk factors, symptoms, Saudi Arabia

## Abstract

**Background:**

Lung Cancer (LC) remains one of the leading causes of cancer-related deaths globally, primarily due to late-stage diagnosis and inadequate public awareness of early symptoms and available screening methods. In Saudi Arabia, rising smoking rates and environmental risks increase the burden, particularly in regions like Asir. This study assesses public knowledge and awareness of LC symptoms, risk factors, and screening practices in the Asir region.

**Methods:**

A cross-sectional online survey was conducted from April to June 2025 among adults (≥18 years) in the Asir region, Saudi Arabia. Using convenience sampling, 437 participants completed a validated Arabic questionnaire assessing demographics, knowledge of LC symptoms and risk factors, and awareness of screening practices. Multivariable logistic regression identified factors significantly associated with knowledge levels (*p* < 0.05).

**Results:**

Out of 437 participants, only 192 (43.9%) demonstrated good knowledge of LC, while the majority 245 (56.1%) had poor knowledge. Symptom awareness varied, with shortness of breath 277 (63.5%) most commonly identified, while frequent chest infections 198 (45.3%) and shoulder pain 129 (29.5%) were less recognized. Only 212 (48.5%) knew of screening methods, though 267 (61.1%) acknowledged the importance of early detection. Logistic regression showed significantly lower odds of good knowledge among high school graduates (OR = 0.242, 95% CI: 0.133–0.440, *p* < 0.001), diploma holders (OR = 0.120, 95% CI: 0.061–0.230, p < 0.001), and uneducated individuals (OR = 0.435, 95% CI: 0.215–0.870, *p* = 0.020) compared to degree holders. Employed participants (175; 40.0%) were more likely to have good knowledge than students (OR = 5.384, 95% CI: 2.650–10.939, *p* < 0.001). Those with smoker exposure among family/friends 228 (52.2%) had lower knowledge (OR = 0.382, 95% CI: 0.237–0.613, *p* < 0.001).

**Conclusion:**

This study highlights insufficient public knowledge of LC in the Asir region, with only 43.9% demonstrating good awareness. While smoking was widely recognized as a major risk factor, awareness of asbestos exposure, chronic obstructive pulmonary disease (COPD), and environmental pollutants was limited, and recognition of less common symptoms was often poor. Knowledge levels were significantly influenced by education, employment, and exposure to smokers. The findings highlight a critical need for targeted educational campaigns and awareness initiatives, particularly among less educated and high-risk populations, to promote early detection and reduce LC burden in the region.

## 1 Introduction

Lung cancer (LC) is a leading global health concern, ranking among the most common malignancies and the primary cause of cancer-related mortality worldwide (1, 2). In 2020 alone, approximately 2.2 million new cases and 1.8 million deaths were reported globally (3, 4). Despite advancements in diagnosis and treatment, LC is often detected at advanced stages, contributing to a five-year survival rate as low as 5% (1).

In Saudi Arabia, LC ranks as the fifth most diagnosed cancer and the third leading cause of cancer-related death, with incidence rates nearly tripling between 1990 and 2016 (5, 6). Projections indicate that by 2025, the country will record over 150,000 new cancer cases and nearly 31,000 deaths, with LC continuing to contribute significantly (7). Despite the growing burden, public awareness and screening practices for LC remain poorly understood, especially in regions like Asir, where no large-scale awareness studies have been conducted. Cultural perceptions, smoking behavior, stigma, and occupational exposures often delay diagnosis and hinder adoption of preventive measures, highlighting the need for region-specific health promotion tailored to the Asir region’s unique sociocultural and environmental profile.

Low awareness of LC symptoms such as persistent cough, haemoptysis (coughing up blood), dyspnoea (shortness of breath), and chest discomfort contributes to delayed healthcare seeking behavior (8–10). Smoking remains the leading risk factor, although 10% of LC cases are unrelated to tobacco use (11). Additional risk factors include occupational exposure to asbestos, arsenic, diesel exhaust, silica, chromium, secondhand smoke, family history, chronic respiratory conditions, and prior radiation therapy (12–14). National and global guidelines emphasize smoking cessation to mitigate these risks (15, 16).

Public awareness of lung cancer remains insufficient. A national survey in Saudi Arabia reported that only 33.6% of respondents were well-informed about cancer and its screening, while just 8.1% had ever undergone any form of screening (17). Moreover, only 31% of primary care physicians were aware of eligibility criteria for LC screening (18). In the southern regions, such as Jazan, research has linked lung cancer risk to respiratory conditions and environmental pollutants (6, 19). Nevertheless, the country still lacks systematic LC screening programs, even though the disease contributes significantly to cancer-related mortality. Despite the high disease burden, current literature reveals a dearth of regional assessments, particularly in the southern regions such as Asir, where environmental and behavioral risk factors may differ. Internationally, low-dose computed tomography (LDCT) has demonstrated success in reducing LC mortality, with the National Lung Screening Trial (NLST) showing a 20% reduction among high-risk individuals (20–22). However, Saudi Arabia does not yet implement nationwide LC screening programs, as outlined by the Saudi National Cancer Centre and Saudi Lung Cancer Association (16). International lung cancer screening guidelines, such as those from the NLST, primarily target high-risk populations based on age and smoking history. However, evidence from Asian populations indicates that these criteria may not fully capture individuals at risk. A study by Wu et al. (23) in Taiwan found that only a small proportion of participants met the NLST criteria, yet a substantial number of lung cancer cases occurred among those who did not meet these guidelines, particularly among women and non-smokers. This highlights the limitations of universal screening criteria and underscores the importance of developing region-specific, risk-based eligibility criteria that account for local demographics, environmental exposures, and the prevalence of non-smoking-related lung cancer. In the context of the Asir region, such tailored criteria are critical to ensure effective early detection and targeted preventive interventions. Although national studies have assessed LC burden and mortality (24, 25) public knowledge and awareness of LC in the Asir region remain underexplored. This gap limits the design of effective awareness and early detection strategies.

The Asir region was selected for this study due to its distinct demographic, environmental, and cultural characteristics that may influence lung cancer risk and awareness. Its mountainous geography, high altitude, and frequent use of biomass fuel contribute to environmental exposures linked to respiratory diseases. Moreover, variations in education, health literacy, and healthcare access can affect awareness and screening behavior. Despite these factors, no prior studies have assessed public awareness of lung cancer in this region, unlike other major Saudi cities. Addressing this gap is essential for developing tailored awareness and early detection strategies suited to the Asir population. Understanding regional awareness and behavioral patterns is essential to guide the development of targeted public health initiatives.

This cross-sectional study aims to assess the level of public knowledge and awareness regarding lung cancer symptoms, risk factors, and screening practices among residents of the Asir region. The findings will help identify knowledge deficits and inform tailored public health interventions aimed at improving early detection and reducing mortality.

## 2 Methods

### 2.1 Study design, setting and participants

This online cross-sectional observational study was conducted between April and June 2025 in the Asir region of Saudi Arabia. The study population included all individuals aged 18 years or older, regardless of gender, who voluntarily consented to participate and were able to understand and independently complete the survey. We excluded incomplete surveys from the analysis. Individuals who did not provide consent, resided outside the Asir region, or were under the age of 18 were not eligible to participate in the study.

### 2.2 Sample size and sampling methods

A convenience sampling method was employed due to the online nature of the survey and ease of access to the general population. Based on the Asir region’s estimated population of 2,024,284 (2011–2022), the minimum required sample size was calculated using the Raosoft sample size calculator. Assuming a 95% confidence level, 5% margin of error, and 50% response distribution, the required sample size was 385 participants. Ultimately, 437 individuals who met the inclusion criteria and completed the survey in full were included in the final analysis.

### 2.3 Data collection and study tool

#### 2.3.1 Questionnaire development and content

Data were collected using a structured, self-administered questionnaire developed after an extensive review of existing literature and input from subject matter experts (24, 25). The questionnaire was initially created in English and then translated into Arabic by two native speakers to ensure linguistic and cultural appropriateness. A reverse translation was performed by an independent translator to ensure accuracy and preserve the original meaning. Discrepancies were resolved through consensus. The final Arabic questionnaire consisted of three main sections: Section 1: Sociodemographic data, including age, gender, nationality, marital status, education level, occupation, monthly income, smoking status, intention to quit smoking (if applicable), and exposure to smokers among family or friends. Section 2: Assessment of LC knowledge, including six general knowledge questions, eight questions on risk factors, and seven on symptoms. Section 3: Six questions evaluating awareness of LC screening and early detection, along with additional questions exploring determinants influencing participants’ decisions to undergo screening.

#### 2.3.2 Tool validation and reliability

A pilot study involving 50 individuals from the target population was conducted to assess the clarity, cultural relevance, and reliability of the instrument. Internal consistency was evaluated using Cronbach’s alpha, which yielded a coefficient of 0.87, indicating good reliability. Based on feedback from the pilot study, minor modifications were made to the wording and order of items. The finalized online questionnaire was distributed electronically via a secure URL.

### 2.4 Knowledge scoring system

Participants’ knowledge of LC was assessed using 21 questions across the domains of general knowledge, risk factors, and symptoms. Each correct answer received one point, while incorrect or “don’t know” responses received zero points. The total knowledge score ranged from 0 to 21. Based on previous literature and expert consultation, a score of ≥11 was considered indicative of good knowledge, while a score <11 was classified as poor knowledge (24).

### 2.5 Recruitment process

Participants were recruited using a combination of in-person and online strategies. Local health facilities including primary care centers, clinics, and Al-Dawaa Pharmacies supported recruitment by displaying QR codes and distributing survey links to walk-in clients. Additionally, the survey link was disseminated via social media platforms such as WhatsApp, Twitter, and Facebook, as well as through community networks across the Asir region. Interested individuals received detailed information regarding the study’s purpose, inclusion criteria, and the voluntary nature of participation. An electronic informed consent form was required before accessing the questionnaire, and only those who provided consent were able to proceed. To ensure data integrity, platform settings were configured to restrict multiple submissions from the same device. Although no personal identifiers were collected, duplicate responses were minimized through technical safeguards.

### 2.6 Ethical approval

The study was conducted in accordance with the principles of the Declaration of Helsinki and received ethical approval from the Institutional Review Board of the King Khalid University Ethics Committee (Approval No. ECM 2025-707). Participants were fully informed about the study’s objectives and assured that all data would be stored securely and treated with strict confidentiality. Informed consent was obtained electronically. Participants indicated their agreement by selecting “I agree to participate” before accessing the questionnaire, and those who declined were unable to proceed. To maintain anonymity, no personally identifiable information was collected, and all responses were analyzed in aggregate. All methods were conducted in accordance with relevant guidelines and regulations.

### 2.7 Statistical analysis

Data analysis was carried out using IBM SPSS Statistics version 23.0. Descriptive statistics, including frequencies and percentages, were used to present categorical variables. Multivariable logistic regression was used to identify factors associated with LC knowledge levels. A *p* < 0.05 was considered statistically significant. Incomplete responses with missing data were excluded from the analysis to maintain the accuracy and integrity of the dataset.

## 3 Results

A total of 437 participants completed the survey on LC screening awareness in the Asir region. The majority were middle-aged adults between 41 and 60 years: 206 (47.14%), followed closely by younger adults aged 18–40 years: 201 (46.00%), while a smaller proportion were aged 61 years or older: 30 (6.86%). Females made up a larger portion of the sample: 259 (59.27%), compared to males: 178 (40.73%). More than half of the participants were married: 246 (56.29%), while 191 (43.71%) were unmarried, divorced, or widowed. In terms of education, 135 (30.89%) held a bachelor’s or master’s degree, 136 (31.12%) had completed high school, 98 (22.43%) held a diploma, and 68 (15.56%) were uneducated. Employment status was nearly evenly distributed, with 175 (40.05%) employed, 176 (40.27%) unemployed, and 86 (19.68%) being college students. Monthly income data showed that a large proportion, 258 (59.04%), did not report their earnings. Among those who did, most earned between 5,000 and 20,000 SAR: 92 (21.05%), followed by 42 (9.61%) earning 20,000–30,000 SAR, 24 (5.49%) earning less than 5,000 SAR, and 21 (4.81%) earning above 30,000 SAR. Regarding smoking behavior, 92 (21.05%) were current smokers, 62 (14.19%) were ex-smokers, and 283 (64.76%) were non-smokers. Among current smokers, only 26 (5.95%) expressed an intention to quit, while 66 (15.10%) did not. Moreover, 228 (52.17%) reported having family members or friends who smoke, while 204 (46.68%) did not (Table 1).

**Table 1:** Sociodemographic characteristics of the study population.

Variables	Number (%)
Age	
18–40 years	201 (46.00)
41–60 years	206 (47.14)
>61 years and older	30 (6.86)
Gender	
Male	178 (40.73)
Female	259 (59.27)
Marital status	
Married	246 (56.29)
Unmarried/ Divorced/Widowed	191 (43.71)
Educational level	
High school	136 (31.12)
Diploma	98 (22.43)
Bachelor/Master degree	135 (30.89)
Uneducated	68 (15.56)
Occupation	
Employed	175 (40.05)
Unemployed	176 (40.27)
Student in colleges	86 (19.68)
Monthly Income	
Less than 5,000 SAR	24 (5.49)
5,000 to 20,000 SAR	92 (21.05)
20,000 SAR to 30,000 SAR	42 (9.61)
Above 30,000 SAR	21 (4.81)
NA	258 (59.04)
Smoking Profile Do you currently smoke?	
Yes	92 (21.05)
No	283 (64.76)
Ex-Smoker	62 (14.19)
If you are a smoker, do you intend to quit?	
Yes	26 (5.95)
No	66 (15.10)
Are any of your family members or friends smokers?	
Yes	228 (52.17)
No	204 (46.68)

The knowledge scores of the participants regarding LC are presented in Table 2. Out of the total 437 respondents, 192 (43.9%) demonstrated good knowledge, while a larger proportion, 245 (56.1%), exhibited poor knowledge. Table 3 summarizes the participants’ general knowledge regarding LC. A total of 241 (55.15%) respondents were aware that LC is among the most common types of cancer, while 182 (41.65%) were not aware, and 14 (3.20%) responded with “I do not know.” Similarly, 255 (58.35%) participants correctly identified LC as one of the leading causes of death, whereas 174 (39.82%) were unaware, and 9 (2.06%) were uncertain. Regarding the misconception of LC being infectious, the majority of participants 304 (69.57%) correctly responded “False,” indicating that it is not an infectious disease. However, 121 (27.69%) incorrectly believed it to be infectious, and 12 (2.75%) were unsure. When asked whether LC is a genetic disease, 146 (33.41%) participants responded “Yes,” while 270 (61.78%) answered “No,” and 21 (4.81%) were unsure. Participants were nearly equally divided in their beliefs about whether a family history of cancer increases the risk of developing it: 209 (47.83%) answered “Yes,” 210 (48.05%) answered “No,” and 18 (4.12%) responded “I do not know.” Regarding early screening for LC, 232 (53.09%) believed that both men and women should undergo screening, while 137 (31.35%) felt it should be limited to smokers only, and 68 (15.56%) believed that only men should be screened.

**Table 2:** Distribution of participants according to lung cancer knowledge scores.

Knowledge	Number (%)
Good	192 (43.9)
Poor	245 (56.1)

**Table 3:** General knowledge of lung cancer among the study population.

Questions	Response	Number (%)
Were you aware that lung cancer is among the most common types of cancer?	Yes	241 (55.15)
	No	182 (41.65)
	I do not know	14 (3.20)
Did you know that lung cancer is one of the leading causes of death?	Yes	255 (58.35)
	No	174 (39.82)
	I do not know	9 (2.06)
Is lung cancer considered an infectious disease?	True	121 (27.69)
	False (Correct Answer)	304 (69.57)
	I do not know	12 (2.75)
In your opinion, is lung cancer a genetic disease?	Yes	146 (33.41)
	No	270
	I do not know	21 (4.81)
Do you believe that having a family history of cancer increases the risk of developing it?	Yes	209 (47.83)
	No	210 (48.05)
	I do not know	18 (4.12)
Who do you think should undergo early screening for lung cancer?	Men only	68 (15.56)
	Both men and women	232 (53.09)
	Smokers only	137 (31.35)

The findings from Table 4 reveal that while the majority of participants demonstrated good awareness of primary risk factors for LC such as smoking 317 (72.54%), shisha use 296 (67.73%), and e-cigarette use 271 (62.01%) awareness was comparatively lower for other contributors like air pollution 218 (49.89%), passive smoking 248 (56.75%), asbestos exposure 226 (51.72%), chronic obstructive pulmonary disease (COPD) 195 (44.62%), and alcohol consumption 193 (44.16%). In terms of symptom recognition, shortness of breath 277 (63.48%) was the most widely identified symptom, followed by chest pain 206 (47.14%), hemoptysis 202 (48.24%), and persistent cough 204 (46.68%). However, fewer participants recognized wheezing 159 (40.96%), frequent chest infections 198 (45.31%), and continuous shoulder pain 129 (29.52%) as potential indicators of LC. These findings highlight both strengths and gaps in public knowledge, emphasizing the need for targeted education on lesser-known environmental, occupational, and clinical warning signs.

**Table 4:** Awareness of risk factors for lung cancer among the study population.

Questions	Response	Number (%)
Is smoking considered a risk factor for lung cancer?	Yes	317 (72.54)
	No	120 (27.46)
	I do not know	3 (0.69)
Do you think that using shisha (water pipe smoking) can increase the risk of lung cancer?	Yes	296 (67.73)
	No	130 (29.75)
	I do not know	11 (2.52)
In your opinion, does smoking e-cigarettes pose a risk for lung cancer?	Yes	271 (62.01)
	No	132 (30.21)
	I do not know	34 (7.78)
Do you believe that exposure to air pollution increases the risk of lung cancer?	Yes	218 (49.89)
	No	155 (35.47)
	I do not know	64 (14.65)
Is passive smoking (secondhand smoke) considered a risk factor for lung cancer?	Yes	248 (56.75)
	No	148 (33.87)
	I do not know	41 (9.38)
Do you think that being exposed to asbestos increases the risk of developing lung cancer?	Yes	226 (51.72)
	No	138 (31.58)
	I do not know	73 (16.70)
In your opinion, is Chronic Obstructive Pulmonary Disease (COPD) associated with an increased risk of lung cancer?	Yes	195 (44.62)
	No	151 (34.55)
	I do not know	91 (20.82)
Do you believe that consuming alcohol is a risk factor for lung cancer?	Yes	193 (44.16)
	No	161 (36.84)
	I do not know	83 (18.99)
Symptoms of lung cancer		
Could chest pain be a warning sign of lung cancer?	Yes	206 (47.14)
	No	179 (40.96)
	Do not Know	52 (11.90)
Is shortness of breath (dyspnea) commonly linked to lung cancer?	Yes	277 (27.46)
	No	120 (9.15)
	Do not Know	40 (36.38)
Might wheezing be a potential indicator of lung cancer?	Yes	159 (40.96)
	No	179 (22.65)
	Do not Know	99 (46.22)
Is coughing up blood (hemoptysis) considered a symptom of lung cancer?	Yes	202 (30.21)
	No	132 (23.57)
	Do not Know	103 (29.75)
Are frequent chest infections a possible indication of lung cancer?	Yes	198 (45.31)
	No	171 (39.13)
	Do not Know	68 (15.56)
Can a persistent cough suggest the presence of lung cancer?	Yes	204 (46.68)
	No	155 (35.47)
	Do not Know	78 (17.85)
Could continuous shoulder pain be a sign of lung cancer?	Yes	129 (29.52)
	No	174 (39.82)
	Do not Know	134 (30.66)

The findings from Table 5 highlight key aspects of public awareness and decision-making regarding LC screening and early detection. While a majority of participants 267 (61.10%) recognized the importance of early detection in saving lives or preventing disease progression, less than half 212 (48.51%) were aware that screening methods for early detection exist. Among those aware, 153 (35.01%) believed that the tests would be beneficial. Over half of the respondents 235 (53.78%) expressed willingness to undergo screening in the future. The leading motivator for screening was the belief that early detection improves prognosis 203 (46.45%), followed by a perceived high personal risk 142 (32.50%), and the perception of high LC rates in Saudi Arabia 115 (26.33%). On the other hand, barriers to screening included the belief of not being at risk 149 (34.54%), fear of a cancer diagnosis 134 (31.02%), and concerns about pain or discomfort from the procedure 126 (29.21%). These results point to moderate awareness and mixed perceptions, underscoring the need for improved education and reassurance regarding the benefits and safety of LC screening.

**Table 5:** Awareness of lung cancer screening and early detection among the study population.

Questions	Response	Number (%)
Do you believe that early detection of lung cancer can help save lives or prevent the disease from advancing?	Yes	267 (61.10)
	No	165 (37.76)
	I do not know	5 (1.14)
Are you aware that screening methods exist for the early detection of lung cancer?	Yes	212 (48.51)
	No	172 (39.36)
	I do not know	53 (12.13)
If you responded “Yes” to the previous question, do you believe that taking these tests would be beneficial?	Yes	153 (35.01)
	No	36 (8.24)
	I do not know	23 (5.26)
Determinants of the decision to undergo lung cancer screening		
Do you plan to undergo lung cancer screening in the future?	Yes	235 (53.78)
	No	202 (46.22)
If you answered “Yes,” what are your reasons for intending to seek lung cancer screening?	Yes *n* (%)	No *n* (%)
A family history of lung cancer	100	135 (30.89)
Being a passive smoker	72	163 (37.30)
A belief that you are at high risk for lung cancer	142	93 (21.28)
A perception that lung cancer rates in Saudi Arabia are high	115	120 (27.46)
A belief that early detection leads to a better prognosis	203	32 (7.32)
If you answered “No,” what are your reasons for intending to seek lung cancer screening?		
Fear of discovering a cancer diagnosis	134	68 (15.56)
Not considering lung cancer a significant health risk	66	137 (31.35)
Concern that the screening procedure may cause pain or discomfort	126	76 (17.39)
A belief that you are not at high risk for lung cancer	149	53 (12.13)

Multivariable logistic regression analysis identified several significant predictors of LC knowledge among participants in the Asir region (Table 6). Education level and occupation were found to be statistically significant. Participants who had completed high school were 75.8% less likely to have good LC knowledge compared to those with a Bachelor/Master’s degree (OR = 0.242; 95% CI: 0.133–0.440; *p* < 0.001), while those with a diploma were 88% less likely (OR = 0.120; 95% CI: 0.061–0.230; *p* < 0.001). Similarly, uneducated participants had significantly lower odds of good knowledge compared to the reference group (OR = 0.435; 95% CI: 0.215–0.870; *p* = 0.020). Regarding occupation, participants who were employed had significantly higher odds of better knowledge compared to college students (OR = 5.384; 95% CI: 2.650–10.939; *p* < 0.001). Employed participants had significantly higher odds of good lung cancer knowledge compared to students, while unemployed status and exposure to smokers in family or social circles were not significantly associated with knowledge. Age, gender, marital status, monthly income, and personal smoking status were not significantly associated with LC knowledge (*p* > 0.05). This suggests that awareness gaps were relatively uniform across age groups and sexes, and were not strongly influenced by marital or economic status. Interestingly, even current or former smokers did not show greater knowledge, highlighting the need for broad-based public health interventions rather than targeting specific demographic or behavioral subgroups. Overall, lower educational attainment and student status were the strongest predictors of reduced lung cancer knowledge, whereas other sociodemographic variables such as age, gender, and smoking status were not significantly associated.

**Table 6:** Multivariable logistic regression analysis of sociodemographic variables associated with lung cancer knowledge scores.

Independent variables	Groups	B value	S. E.	Wald	df	*p*-value	OR	95% C. I. for OR	
								Lower	Upper
Age	18–40 years	Reference							
	41–60 years	0.107	0.273	0.154	1	0.695	1.113	0.652	1.900
	61 years and older	1.007	0.595	2.867	1	0.090	2.737	0.853	8.777
Gender	Male	Reference							
	Female	−0.304	0.255	1.424	1	0.233	0.738	0.448	1.216
Marital status	Married	Reference							
	Unmarried/Divorced/Widowed	−0.339	0.270	1.580	1	0.209	0.712	0.420	1.209
Education	High school	−1.418	0.305	21.661	1	*0.000	0.242	0.133	0.44
	Diploma	−2.122	0.346	37.659	1	*0.000	0.120	0.061	0.23
	Bachelor/Master degree	Reference							
	Uneducated	−0.832	0.359	5.382	1	*0.020	0.435	0.215	0.87
Occupation	Employed	1.683	0.362	21.660	1	*0.000	5.384	2.650	10.939
	Unemployed	0.365	0.593	0.379	1	0.538	1.441	0.450	4.611
	Student in colleges	Reference							
Monthly income	Less than 5,000 SAR	1.604	0.927	2.997	1	0.083	4.973	0.809	30.574
	5,000 to 20,000 SAR	0.206	0.805	0.065	1	0.798	1.228	0.254	5.947
	20,000 SAR to 30,000 SAR	0.241	0.842	0.082	1	0.775	1.272	0.244	6.627
	Above 30,000 SAR	Reference							
	NA	0.326	0.602	0.293	1	0.588	1.386	0.426	4.509
Do you currently smoke?	Yes	−0.356	0.296	1.444	1	0.229	0.701	0.392	1.252
	No	Reference							
	Ex-smoker	−0.026	0.346	0.005	1	0.941	0.975	0.495	1.919
Are any of your family members or friends smokers?	Yes	−0.964	0.242	15.830	1	*0.000	0.382	0.237	0.613
	No	Reference							
	Constant	−1.085	0.707	2.356	1	0.125	0.338		

*Indicates *P* < 0.05, S.E.: Standard error, B value: Beta value, CI: Confidence interval.

## 4 Discussion

This cross-sectional study assessed public knowledge and awareness of LC and its screening practices among 437 individuals in the Asir region, Saudi Arabia. The sample was predominantly aged 18–60 years, with a higher proportion of females (59.27%). Educational levels varied, with 15.56% uneducated and over 30% holding university degrees. Notably, 21.05% were current smokers, yet only 5.95% intended to quit, while 52.17% reported secondhand smoke exposure, emphasizing the need for targeted tobacco control efforts. Overall, only 43.9% of respondents demonstrated good knowledge of LC, consistent with findings from Jazan, Saudi Arabia, where moderate awareness and poor knowledge were reported (25, 26). Awareness appears higher in some international populations, such as 79.7% in one study (24) and over 50% among tertiary students in Malaysia (19), likely reflecting differences in education, healthcare access, and public health campaigns. These findings highlight persistent gaps in LC knowledge in the Asir region, which may contribute to delays in recognition, diagnosis, and treatment.

Regarding smoking behavior, 21.05% of participants were current smokers, 14.19% were ex-smokers, and 64.76% were non-smokers. The low intention to quit despite widespread secondhand smoke exposure highlights a significant public health concern, exceeding prior local estimates (25) and aligning with global prevalence (27). Secondhand smoke exposure was reported by 56.75% of participants, emphasizing the need for interventions addressing both active and passive tobacco exposure (28, 29). In comparison, only 49.2% of adults over 40 in an Australian study recognized secondhand smoke as a risk, with limited awareness of the benefits of quitting smoking (30). These findings underscore the importance of culturally sensitive smoking cessation initiatives, public health campaigns targeting social norms, and community-based programs to reduce tobacco exposure. Awareness of other LC risk factors varied. While 72.54% recognized smoking as a major risk factor, knowledge of shisha (67.73%), e-cigarette use (62.01%), asbestos exposure (51.72%), and chronic obstructive pulmonary disease (COPD) (44.62%) was lower, though higher than in some previous studies (25, 26). The relatively higher awareness of occupational hazards like asbestos exposure suggests some success of ongoing regional awareness campaigns; however, broader education on environmental pollutants, genetic predisposition, and chronic respiratory conditions remains necessary to foster a more comprehensive understanding of LC risk.

Symptom recognition was highest for shortness of breath (63.48%), hemoptysis (48.24%), chest pain (47.14%), and persistent cough (46.68%), with lower recognition for wheezing (40.96%), frequent chest infections (45.31%), and continuous shoulder pain (29.52%). Compared to other studies, symptom awareness was relatively low (26, 31–33), reinforcing the need for region-specific campaigns to educate the public on both common and less emphasized LC symptoms. Enhanced symptom awareness could promote earlier medical consultation, timely diagnosis, and improved prognosis. Awareness of LC screening was suboptimal: only 48.51% were aware of screening methods, and just 35.01% believed these tests to be beneficial. Common barriers included fear of diagnosis, perceived discomfort, and low perceived risk, consistent with findings from London and other regions (34–36). Regression analysis identified education and occupation as significant predictors of LC knowledge, whereas age, gender, marital status, income, and smoking status were not, highlighting the importance of context-specific, sociodemographically tailored strategies (24, 37–40). Notably, employed participants demonstrated significantly higher knowledge compared to students, while being unemployed or having exposure to smokers in family or social circles did not significantly affect knowledge levels. These findings highlight that limited education and unemployment contribute substantially to poor awareness of lung cancer in the Asir region. Therefore, targeted public health initiatives should focus on reaching less educated and student populations through accessible, culturally relevant educational campaigns, community workshops, and workplace health programs. These results suggest that interventions targeting less educated and student populations could be particularly impactful.

Encouragingly, 53.78% expressed willingness to undergo LC screening, motivated by belief in early detection (86.38%) and perceived personal risk (60.43%). However, nearly half were unwilling, citing fear, discomfort, and low perceived susceptibility, emphasizing the need for interventions addressing misconceptions, stigma, and fatalistic attitudes toward cancer (41, 42). Social perceptions that smoking is a lifestyle choice rather than an addiction may further hinder engagement with preventive services. Integrating empathetic, culturally sensitive messaging into public health campaigns may help overcome these barriers and improve screening uptake.

Recent research highlights the importance of considering gender differences and East–West cultural variations in lung cancer risk and screening strategies. In Western countries, screening guidelines focus primarily on smoking history, potentially overlooking nonsmokers at risk from environmental exposures or genetic factors. In contrast, Asian populations, including Saudi Arabia, show an increasing incidence of LC among nonsmokers, particularly women. Cultural norms and gender roles may influence health-seeking behavior, with women in certain societies experiencing barriers to accessing preventive services or participating in screening programs (43). These findings suggest that culturally tailored interventions addressing gender-specific barriers are essential. Strategies may include community engagement, educational campaigns targeting both men and women, and healthcare provider training to recognize and mitigate gender-related disparities. Integrating such considerations into LC screening programs could enhance early detection and improve outcomes across populations (43).

Overall, these findings highlight several actionable points for policy and practice. First, culturally appropriate educational campaigns should target both common and less recognized LC symptoms, risk factors, and the benefits of early detection. Second, healthcare providers should be trained in standardized communication strategies to address patient fears, misconceptions, and stigma. Third, leveraging primary care, community hubs such as schools, mosques, and workplaces, and digital platforms could enhance outreach and engagement. Finally, integrating LC awareness into broader non-communicable disease initiatives can strengthen preventive health infrastructure and support Saudi Arabia’s Vision 2030 public health goals. By addressing gaps in knowledge and screening uptake, these strategies can ultimately contribute to earlier diagnosis, improved clinical outcomes, and reduced LC-related morbidity and mortality in the Asir region.

### 4.1 Limitations

This study has several limitations. First, the cross-sectional design captures only a snapshot in time, preventing causal inferences between knowledge levels and associated factors. Second, data were self-reported, particularly for smoking behavior, which may be subject to recall and social desirability bias. Third, the study was conducted in a single region (Asir), which may limit the generalizability of findings to other areas of Saudi Arabia with differing socio-cultural and healthcare contexts. Fourth, the online survey method may have excluded individuals without internet access or adequate digital literacy, potentially biasing the sample toward more educated respondents. Fifth, a large proportion of participants (59.04%) did not report their monthly income, likely because many were college students or unemployed, thereby limiting the assessment of socioeconomic influences on LC knowledge. However, the convenience sampling method, while practical and suitable for online surveys, may limit the generalizability of the findings. Participants were self-selected and may represent individuals who are more health-conscious or technologically literate, potentially introducing selection bias. Therefore, the results should be interpreted with caution and may not fully reflect the awareness and knowledge of the entire Asir region population. Finally, although the questionnaire captured awareness, knowledge, and attitudes, it did not assess behavioral intent or actual participation in screening programs. As a result, the study cannot fully evaluate the translation of knowledge into preventive actions. Future research should include measures of screening behavior and intentions to better understand the knowledge-to-action gap and inform interventions aimed at increasing participation in early detection programs.

## 5 Conclusion

This study reveals a significant gap in public knowledge of LC in the Asir region, with only 43.9% of participants demonstrating good knowledge and fewer than half aware of available screening methods. While smoking was widely recognized as a major risk factor, awareness of other contributors such as asbestos exposure, COPD, and environmental pollutants was limited. Multivariable regression analysis identified education level, employment status, and exposure to smokers as significant predictors of knowledge. Participants with higher education and those employed demonstrated greater awareness, whereas those regularly exposed to smokers had significantly lower knowledge levels.

These findings underscore the need for targeted, community based education initiatives to correct misconceptions, improve symptom recognition, and encourage early screening. Future studies should use more representative sampling strategies and consider longitudinal or qualitative methods to explore knowledge trends and sociocultural influences. Public health interventions must emphasize the risks of secondhand smoke and support smoking cessation efforts. Ultimately, large-scale, nationwide lung cancer awareness campaigns particularly those focused on high-risk and underserved populations are essential for promoting early detection, reducing the disease burden, and improving lung cancer outcomes in Saudi Arabia.

## Data Availability

The original contributions presented in the study are included in the article/supplementary material, further inquiries can be directed to the corresponding author.

## References

[1] Bray F Ferlay J Soerjomataram I Siegel RL Torre LA Jemal A. Global cancer statistics 2018: GLOBOCAN estimates of incidence and mortality worldwide for 36 cancers in 185 countries. CA Cancer J Clin. (2018) 68:394–424. doi: 10.3322/caac.21492, PMID: 30207593

[2] Abraham Z Massawe E Ntunaguzi D Kahinga A Mawala S. Prevalence of noise-induced hearing loss among textile industry workers in dares salaam, Tanzania. Ann Glob Health. (2019) 85:85. doi: 10.5334/aogh.2352, PMID: 31225954 PMC6634445

[3] Sung H Ferlay J Siegel RL Laversanne M Soerjomataram I Jemal A. Global Cancer statistics 2020: GLOBOCAN estimates of incidence and mortality worldwide for 36 cancers in 185 countries. CA Cancer J Clin. (2021) 71:209–49. doi: 10.3322/caac.21660, PMID: 33538338

[4] Barta JA Powell CA Wisnivesky JP. Global epidemiology of LC. Ann Glob Health. (2019) 85:2419. doi: 10.5334/aogh.2419 PMC6724220 30741509

[5] Althubiti MA Eldein MMN. Trends in the incidence and mortality of cancer in Saudi Arabia. Saudi Med J. (2018) 39:1259–62. doi: 10.15537/smj.2018.12.23348, PMID: 30520511 PMC6344657

[6] Almatroudi A. A retrospective cohort study of lung Cancer incidences and epidemiological analysis in Saudi Arabian population from 2006–2016. Int J Environ Res Public Health. (2021) 18:11827. doi: 10.3390/ijerph182211827, PMID: 34831584 PMC8622663

[7] Alattas MT. Cancer control priorities and challenges in Saudi Arabia: a preliminary projection of cancer burden. Gulf J Oncol. (2019) 1:22–30. 30956193

[8] Qadhi OA Alghamdi A Alshael D Alanazi MF Syed W Alsulaihim IN. Knowledge and awareness of warning signs about lung cancer among pharmacy and nursing undergraduates in Riyadh, Saudi Arabia – an observational study. J Cancer. (2023) 14:3378–86. doi: 10.7150/jca.89358, PMID: 38021161 PMC10647201

[9] Beckles MA Spiro SG Colice GL Rudd RM. Initial evaluation of the patient with LC: symptoms, signs, laboratory tests, and paraneoplastic syndromes. Chest. (2003) 123:97S–104S. doi: 10.1378/chest.123.1_suppl.97s 12527569

[10] Simon AE Juszczyk D Smyth N Power E Hiom S Peake MD. Knowledge of LC symptoms and risk factors in the U.K.: development of a measure and results from a population-based survey. Thorax. (2012) 67:426–32. doi: 10.1136/thoraxjnl-2011-200898 22426791

[11] Health, S.M.o. LC; (2022). Available online at: https://www.moh.gov.sa/en/awarenessplateform/ChronicDisease/Pages/LungCancer.aspx (Accessed August 9, 2023)

[12] Malhotra J Malvezzi M Negri E La Vecchia C Boffetta P Tindle HA. Risk factors for LC worldwide. Eur Respir J. (2016) 48:889–902. doi: 10.1183/13993003.00359-2016 27174888

[13] Al-Hamdan N Ravichandran K Al-Sayyad J Al-Lawati J Khazal Z Al-Khateeb F. Incidence of cancer in gulf cooperation council countries, 1998-2001. East Mediterr Health J. (2009) 15:600–11. doi: 10.26719/2009.15.3.600 19731776

[14] Jazieh AR Algwaiz G Alshehri SM Alkattan K. LC in Saudi Arabia. J Thorac Oncol. (2019) 14:957–62. doi: 10.1016/j.jtho.2019.01.023 31122559

[15] Su Z Jia XH Zhao FH Zhou QH Fan YG Qiao YL. Effect of time since smoking cessation on LC incidence: an occupational cohort with 27 follow-up years. Front Oncol. (2022) 12:817045. doi: 10.3389/fonc.2022.817045 35299746 PMC8921458

[16] Jazieh AR AlGhamdi M AlGhanem S AlZahrani M AlAmri A AlMutairi M. Saudi LC prevention and screening guidelines. Ann Thorac Med. (2018) 13:198–204. doi: 10.4103/atm.ATM_147_18 30416590 PMC6196665

[17] Elmaghraby DA Alshalla AA Alyahyan A Altaweel M Al Ben Hamad AM Alhunfoosh KM. Public knowledge, practice, and attitude regarding cancer screening: a community-based study in Saudi Arabia. Int J Environ Res Public Health. (2023) 20:1114. doi: 10.3390/ijerph20021114, PMID: 36673870 PMC9859105

[18] Duong DK Shariff-Marco S Cheng I Naemi H Moy LM Haile R. Patient and primary care provider attitudes and adherence towards LC screening at an academic medical center. Prev Med Rep. (2017) 6:17–22. doi: 10.1016/j.pmedr.2017.01.012 28210538 PMC5304233

[19] Al-Ahmadi K Al-Zahrani A. NO2 and Cancer incidence in Saudi Arabia. Int J Environ Res Public Health. (2013) 10:5844–62. doi: 10.3390/ijerph10115844, PMID: 24192792 PMC3863874

[20] Field JK Vulkan D Davies MPA Baldwin DR Brain KE Devaraj A. LC mortality reduction by LDCT screening: UKLS randomised trial results and international meta-analysis. Lancet Reg Health Eur. (2021) 10:100179. doi: 10.1016/j.lanepe.2021.100179 34806061 PMC8589726

[21] de Koning HJ van der Aalst CM de Jong PA Scholten ET Nackaerts K Heuvelmans MA. Reduced lung-Cancer mortality with volume CT screening in a randomized trial. N Engl J Med. (2020) 382:503–13. doi: 10.1056/NEJMoa1911793, PMID: 31995683

[22] National Lung Screening Trial Research Team Aberle DR Adams AM Berg CD Black WC Clapp JD. Reduced lung-cancer mortality with low-dose computed tomographic screening. N Engl J Med. (2011) 365:395–409. doi: 10.1056/NEJMoa1102873 21714641 PMC4356534

[23] Wu FZ Huang YL Wu CC Tang EK Chen CS Mar GY. Assessment of selection criteria for low-dose lung screening CT among Asian ethnic groups in Taiwan: from mass screening to specific risk-based screening for non-smoker lung Cancer. Clin Lung Cancer. (2016) 17:e45–56. doi: 10.1016/j.cllc.2016.03.004, PMID: 27133540

[24] Alamri SA Alzahrani MM Alamri AA Khalifa WW Alsulami RY Bardesi J. Understanding the public knowledge, attitude, and practice toward screening and risk factors of LC in Saudi Arabia: a cross-sectional study. Ann Thorac Med. (2024) 19:275–83. doi: 10.4103/atm.atm_111_24. 39544349 PMC11559701

[25] Madkhali MA Alhazmi E Hakami F Darraj H Hamdi S Hakami KM. A cross-sectional study on the knowledge and awareness of LC and screening in Jazan region, Saudi Arabia. J Multidiscip Healthc. (2023) 16:3857–70. doi: 10.2147/JMDH.S435129 38076592 PMC10710187

[26] Dlamini SB Sartorius B Ginindza TG. Knowledge, attitudes and practices towards LC among adults in KwaZulu-Natal, South Africa: a cross-sectional survey. J Public Health Afr. (2022) 13:2111. doi: 10.4081/jphia.2022.2111 36313926 PMC9614694

[27] Alison Commar (WHO Jenewa), Vinayak Prasad (WHO Jenewa) ET d’Espaignet (Universitas N, Australia). WHO global report on trends in prevalence of tobacco use. WHO (Geneva, Switzerland: World Health Organization). (2000).

[28] Moradi-Lakeh M El Bcheraoui C Tuffaha M Daoud F Saeedi M Basulaiman M. Tobacco consumption in the Kingdom of Saudi Arabia, 2013: findings from a national survey. BMC Public Health. (2015) 15:611. doi: 10.1186/s12889-015-1902-330, PMID: 26141062 PMC4491232

[29] Couraud S Labonne S Missy P Souquet PJ Cortot AB Cadranel J. BioCAST: le Bio-observatoire national du cancer bronchiques chez les patients non fumeurs (IFCT1002). Rev Mal Respir. (2013) 30:576–83. doi: 10.1016/j.rmr.2013.03.00629 24034464

[30] Crane M Scott N O'hara BJ Aranda S Lafontaine M Stacey I. Knowledge of the signs and symptoms and risk factors of LC in Australia: mixed methods study. BMC Public Health. (2016) 16:508. doi: 10.1186/s12889-016-3051-8 27296668 PMC4906715

[31] Loh JF Tan SL. LC knowledge and screening in the context of the Malaysian population. JPPR J Pharm Pract Res. (2018) 48:56–64. doi: 10.1002/jppr.1341

[32] Shankar A Roy S Malik A Rath GK Julka PK Kamal VK. Level of awareness of various aspects of LC among college teachers in India: impact of cancer awareness programmes in prevention and early detection. J Cancer Educ. (2016) 31:709–23. doi: 10.1007/s13187-015-0960-7 26687206

[33] Al-Naggar RA Kadir S. LC knowledge among secondary school male teachers in Kudat. Asian Pac J Cancer Prev. (2013) 14:103–12. doi: 10.7314/APJCP.2013.14.1.103 23534705

[34] Quaife SL McEwen A Janes SM Wardle J. Attitudes towards LC screening in socioeconomically deprived and heavy smoking communities: informing screening communication. Health Expect. (2017) 20:563–73. doi: 10.1111/hex.12481 27397651 PMC5513004

[35] Lebrett MB Crosbie EJ Yorke J Hewitt K Rowlands A Badrick E. Risk perception and disease knowledge in attendees of a community-based LC screening programme. LC. (2022) 168:1–9. doi: 10.1016/j.lungcan.2022.04.003 35430354

[36] Lowenstein M Vijayaraghavan M Burke NJ Karliner L Wang S Peters M. Real-world LC screening decision-making: barriers and facilitators. LC. (2019) 133:32–7. doi: 10.1016/j.lungcan.2019.04.026 31200824

[37] Thabit H Zainuddin N. Knowledge and perception on LC and its screening: a study among undergraduate students of the International Islamic University Malaysia, Kuantan campus. Biomed Clin Sci. (2017) 2:61–6.

[38] El Rhazi K Bennani B El Fakir S Boly A Bekkali R Zidouh A. Public awareness of cancer risk factors in the Moroccan population: a population-based cross-sectional study. BMC Cancer. (2014) 14:695. doi: 10.1186/1471-2407-14-695, PMID: 25245224 PMC4190496

[39] Viswanath K Finnegan JR. The knowledge gap hypothesis: twenty-five years later. Ann Int Commun Assoc. (1996) 19:187–228. doi: 10.1080/23808985.1996.1167893121

[40] Chen LS Kaphingst KA. Risk perceptions and family history of LC: differences by smoking status. Public Health Genomics. (2011) 14:26–34. doi: 10.1159/00029415122 20375490 PMC3025884

[41] Weiner B Perry RP Magnusson J. An attributional analysis of reactions to stigmas. J Pers Soc Psychol. (1988) 55:738–48. doi: 10.1037//0022-3514.55.5.738, PMID: 2974883

[42] Stuber J Galea S Link BG. Smoking and the emergence of a stigmatized social status. Soc Sci Med. (2008) 67:420–30. doi: 10.1016/j.socscimed.2008.03.010, PMID: 18486291 PMC4006698

[43] Wu FZ Chang J Chen W Li Y Zhang S Wang H. Toward more effective lung cancer risk stratification to empower screening programs for the Asian nonsmoking population. J Am Coll Radiol. (2023) 20:156–61. doi: 10.1016/j.jacr.2022.10.010 36646597

